# La tamponnade cardiaque: une manifestation rare de l'hypothyroïdie

**DOI:** 10.11604/pamj.2015.22.104.7941

**Published:** 2015-10-06

**Authors:** Salim Arous, Youssef Ettaoumi, Hayat Najih, Said Makani, Arroussi Aziz Alami, Rachida Habbal

**Affiliations:** 1 Service de Cardiologie, CHU Ibn Rochd, Casablanca, Maroc; 2 Service de Chirurgie Cardio-Vasculaire, CHU Ibn Rochd, Casablanca, Maroc

**Keywords:** Hypothyroïdie, drainage chirurgical, tamponnade cardiaque, hypothyroidism, surgical drainage, cardiac tamponade

## Abstract

La survenue d'un épanchement péricardique au cours d'une hypothyroïdie est fréquente. Ce fait justifie la réalisation d'une échocardiographie lors du diagnostic et du suivi évolutif des hypothyroïdies. Les signes de mauvaise tolérance de cet épanchement sont rares et la constitution d'une tamponnade péricardique n'est que très rarement rapportée. Ce travail se base sur l’étude rétrospective de quatre cas de tamponnade cardiaque révélant une hypothyroïdie primaire. L’échocardiographie a permis de faire le diagnostic immédiat de la tamponnade. Orienté par l'aspect clinique, le diagnostic d'hypothyroïdie a été confirmé par dosage biologique. Le traitement a été à base de drainage péricardique chirurgical et d'hormonothérapie progressive, l’évolution a été favorable avec disparition de l’épanchement péricardique. Certaines particularités physiopathologiques, l'intérêt de l’échocardiographie et du drainage péricardique chirurgical, tant au plan diagnostique qu'au plan thérapeutique sont soulignés.

## Introduction

L’épanchement péricardique de moyenne ou de grande abondance secondaire à l'hypothyroïdie a été bien décrit. La plupart des cas rapportés ont souligné la rareté de la tamponnade cardiaque associée à une hypothyroïdie, qui est généralement marquée par l'apparition d'une infection virale intercurrente accélérant l’évolution vers la tamponnade [1, 2]. Le diagnostic précoce de l’épanchement péricardique et de son étiologie est important pour améliorer le pronostic et peut éviter les examens inutiles et onéreux [3]. Nous rapportons une série de quatre cas de tamponnade associée à une hypothyroïdie, colligés au pôle cardio-vasculaire du CHU Ibn Rochd Casablanca amenant à discuter les différents aspects de cette affection.

## Patient et observation

**Figure 1:**
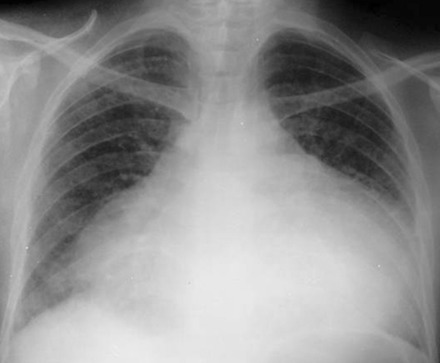
Radiographie du thorax face chez la patiente numéro 1: aspect de cardiomégalie

**Figure 2:**
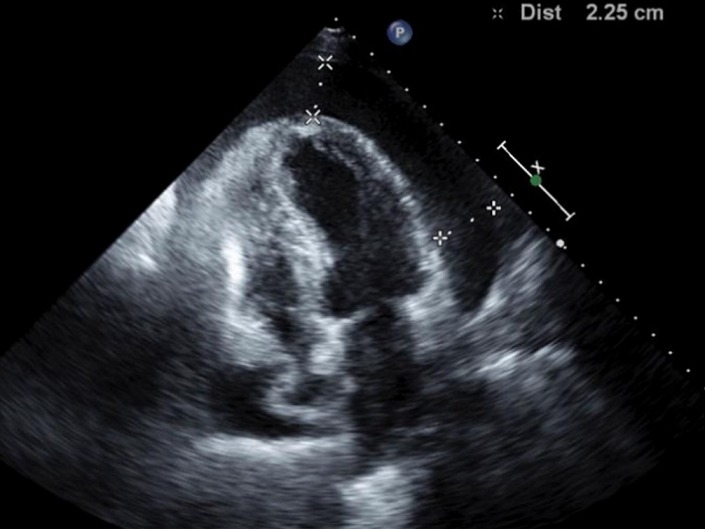
Echocardiographie transthoracique chez la patiente numéro 2: coupe apicale montrant un épanchement péricardique prédominant en regard des cavités gauches

Nous avons traité au pôle cardio-vasculaire du CHU Ibn Rochd Casablanca, 4 cas de tamponnade associée à une hypothyroïdie durant l'année 2010, dont les observations sont résumées dans le Tableau 1. I1 s'agit de quatre femmes d'une moyenne d’âge de 37,5 ans avec un antécédent de syndrome grippal une semaine avant l'hospitalisation chez deux patientes. Les signes d'appel étaient dans tous les cas une dyspnée aggravative devenant au repos, la douleur thoracique était notée deux fois. Cliniquement nos 4 patientes présentaient une hypotension artérielle sans signes d'hypoperfusion périphériques, avec des signes d'insuffisance cardiaque droite. La thyroïde n’était pas palpable chez les 4 patientes. La radiographie du thorax montrait une cardiomégalie dans les quatre cas (Figure 1). Le diagnostic d'hypothyroïdie a été fondé sur les résultats du laboratoire, définit par un taux élevé de TSHus et un taux faible T4 libre. L’échocardiographie trans-thoracique a été réalisée chez toutes les patientes, la présence des critères de gravité a été l'indication à opérer en urgence, en montrant un épanchement péricardique de grande abondance avec des signes compressifs (Figure 2) et des variations respiratoires significatives des flux mitral, aortique et tricuspidien.

**Tableau 1:** Résumé des quatre observations

Observations	N°1	N°2	N°3	N°4
Age	36 ans	44 ans	52 ans	49 ans
Sexe	F	F	F	F
Antécédents d'hypothyroïdie	Non	oui	oui	non
Signes fonctionnels	-Dyspnée stade IV -Douleurs thoraciques	-Dyspnée stade IV -Malaise -Fatigue -Lenteur	-Dyspnée stade IV -Douleurs thoraciques	-Dyspnée stade IV -Angoisse -Malaise -Lenteur
Signes physiques	-Polypnée -Tachycardie à 95 bpm -PA = 92/50mmhg -Pas de pouls paradoxal-Assourdissement des bruits du cœur-Turgescence des veines jugulaires -Thyroïde non palpable	-Polypnée -Tachycardie à 105 bpm -PA = 80/40mmhg -Pas de pouls paradoxal -Assourdissement des bruits du cœur -Turgescence des veines jugulaires -Hépatomégalie -Thyroïde non palpable -Œdèmes des membres inférieurs	-Polypnée -Tachycardie à 98 bpm -PA = 90/40mmhg -Pas de pouls paradoxal -Assourdissement des bruits du cœur -Turgescence des veines jugulaires -Thyroïde non palpable	-Polypnée -Tachycardie à 110 bpm -PA = 90/45mmhg -Pas de pouls paradoxal -Assourdissement des bruits du cœur -Turgescence des veines jugulaires -Thyroïde non palpable
Radio thorax	Cardiomégalie			
ECG	Microvoltage Tachycardie sinusale	Microvoltage Tachycardie sinusale	Microvoltage Tachycardie sinusale	Microvoltage Tachycardie sinusale
Echocardiographie transthoracique	-Epanchement péricardique circonférentiel de 36 mm en regard du ventricule droit et 28 mm en postérieur. -Diminution du flux transmitral et aortique respectivement de 32% et 26% et augmentation du flux tricuspidien de 44% à l'inspiration. -VCI dilatée à 24 mm non compliante.	-Epanchement péricardique circonférentiel prédominant en postérieur (22 mm). - Diminution du flux transmitral et aortique respectivement de 24% et 25% et augmentation du flux tricuspidien de 35% à l'inspiration. - VCI dilatée à 19 mm peu compliante.	-Epanchement péricardique circonférentiel de 22 mm en regard du ventricule droit et 23 mm en postérieur. - Diminution du flux transmitral et aortique respectivement de 27% et 29% et augmentation du flux tricuspidien de 40% à l'inspiration. - VCI dilatée à 22 mm non compliante.	-Epanchement péricardique circonférentiel de 21 mm en regard du ventricule droit et 26 mm en postérieur. - Diminution du flux transmitral et aortique respectivement de 25% et 22% et augmentation du flux tricuspidien de 39% à l'inspiration. - VCI dilatée à 25 mm non compliante.
Dosage des hormones thyroïdiennes	TSHus = 95,84µIU/ml T3 libre = 28ng/dl T4 libre = 0,2ng/dl	TSHus = 207µIU/ml T3 libre = 11ng/dl T4 libre = 0,1ng/dl	TSHus = 156µIU/ml T3 libre = 17ng/dl T4 libre = 0,19ng/dl	TSHus = 194µIU/ml T3 libre = 16ng/dl T4 libre = 0,13ng/dl
Traitement	Drainage péricardique + Hormones thyroïdiennes de synthèse par voie orale			
Evolution	Favorable			

Toutes les patientes ont été opérées en urgence bénéficiant d'un drainage péricardique chirurgical selon la même technique: la péricardotomie sous-xiphoïdienne sous anesthésie locale. Une exploration macroscopique a été faite avec appréciation de l’état du cœur et aspiration d'un liquide jaune citrin, un drain de bon calibre a été laissé sur place, sortant par une autre incision avec réalisation d'une biopsie péricardique dont le résultat, chez nos quatre patientes, était non contributif. En dehors du dosage des hormones thyroidiennes, le reste du bilan étiologique réalisé était négatif, comportant un examen gynécologique, une échographie abdomino-pelvienne, une mammographie, un bilan à la recherche de tuberculose et un bilan de maladies de système. La durée d'hospitalisation a été en moyenne de 7 jours. Nos quatre patientes ont bénéficié d'un traitement hormonal substitutif, l’évolution a été bonne, contrôlées 3 mois après à l’échocardiographie, nous avons noté une absence de récidive de l’épanchement péricardique, et une normalisation du taux de TSHus.

## Discussion

L'hypothyroïdie peut provoquer des épanchements des différentes cavités du corps, y compris le péritoine, le péricarde, la plèvre, l'oreille moyenne, l'uvée, les articulations, et le scrotum [4]. Ces épanchements sont exsudatifs et le mécanisme est principalement l'extravasation de mucopolysaccharide hygroscopique dans les cavités du corps avec augmentation de la perméabilité capillaire, une diminution du drainage lymphatique, et une meilleure rétention de sel et d'eau [2]. L'accumulation du liquide est généralement lente et le péricarde a la capacité à se distendre de façon chronique de sorte que les modifications hémodynamiques sont peu susceptibles d’être présentes même avec un épanchement péricardique massif [3, 5]. Les épanchements chez les patients atteints d'hypothyroïdie ont généralement un taux élevé de cholestérol, la péricardite à cholestérol causant la tamponnade a été rapportée [6]. Bien que l’épanchement péricardique est fréquent dans l'hypothyroïdie, la tamponnade cardiaque a été retrouvée dans la plupart des cas seulement après que le patient a eu des symptômes depuis de nombreuses années ou chez les patients déjà traité pour une hypothyroïdie [1, 3]. La plupart des cas de tamponnade ont été signalés chez les sujets âgés et les sujets de sexe féminin [2]. Il y a eu quelques cas d’épanchement péricardique massif dû à une hypothyroïdie signalés chez l'enfant [7].

Cependant, les patients ayant une hypothyroïdie avec épanchements péricardiques importants peuvent ne pas toujours avoir les signes d'hypothyroïdie, tels que la prise de poids, faiblesse, œdème, lenteur. Ceci a été clairement démontré par deux de nos patientes, qui n'avaient pas les symptômes évidents ou des signes d'hypothyroïdie. Par conséquent, l'hypothyroïdie doit être écartée chez tous les patients avec un épanchement péricardique inexpliqué. Les signes classiques de tamponnade cardiaque (la triade de Beck) sont: l'hypotension artérielle, l’élévation de la pression veineuse centrale et l'assourdissement des bruits cardiaques, des signes qui n’étaient pas toujours présents chez nos patientes. Le pouls paradoxal est habituel, mais pas toujours évident. Le frottement péricardique est habituel avec un épanchement de faible à moyenne abondance [3, 5]. L'ECG peut montrer un microvoltage avec alternance électrique, qui peut être causée soit par un myxœdème ou par un épanchement péricardique. Le diagnostic de l’épanchement péricardique est généralement suspecté par la radiographie du thorax et confirmé par l’échocardiographie, qui est la procédure diagnostique de choix, avec une très haute sensibilité et spécificité. Les signes échocardiographiques de tamponnade, ont été présents chez nos 4 patientes, comprenant le collapsus télédiastolique du ventricule droit, la compression de l'oreillette droite, et une déviation du septum interventriculaire dans le ventricule gauche à l'inspiration. Avec un traitement médical adéquat de l'hypothyroïdie par les hormones thyroïdiennes et les stéroïdes, la grande majorité des épanchements péricardiques se résorbent lentement mais complètement et rarement la chirurgie est nécessaire [1–3]. La péricardocentèse ou une intervention chirurgicale ne sont nécessaire que dans les cas de tamponnade péricardique [2, 5]. La survenue d'une tamponnade cardiaque en cas d'hypothyroïdie est très rare en raison de la lenteur d'accumulation du liquide et de la distensibilité péricardique. Quand une tamponnade se produit, elle peut être causée par des facteurs provoquant tels une péricardite virale concomitante [3, 8].

## Conclusion

L'hypothyroïdie doit être écartée chez tous les patients avec un épanchement péricardique inexpliqué, non seulement chez les patients atteints d'hypothyroïdie manifeste cliniquement ou chez les personnes âgées. Enfin, une fois le diagnostic a été établi, le traitement avec des hormones thyroïdiennes conduit généralement à la résolution de l’épanchement péricardique pendant 2-12 mois sans séquelles.
